# Associations of Metabolic Syndrome and Insulin Resistance With Attenuated Executive Function Post‐Preeclampsia: A Nested Case–Control Study

**DOI:** 10.1111/1471-0528.18186

**Published:** 2025-04-29

**Authors:** Robert‐Jan Alers, Chahinda Ghossein‐Doha, Yentl Brandt, M. Eline Kooi, Suzanne C. Gerretsen, Jacobus F. A. Jansen, Walter H. Backes, Vincent van de Ven, Petra P. M. Hurks, Marc E. A. Spaanderman

**Affiliations:** ^1^ Department of Obstetrics and Gynaecology, Maastricht University Medical Centre+ (MUMC+) Maastricht the Netherlands; ^2^ GROW, School for Oncology and Developmental Biology, Maastricht University Maastricht the Netherlands; ^3^ Cardiovascular Institute, Thorax Centre, Department of Cardiology Erasmus Medical Centre Rotterdam the Netherlands; ^4^ CARIM, School for Cardiovascular Diseases, Maastricht University Maastricht the Netherlands; ^5^ Department of Radiology and Nuclear Medicine MUMC+ Maastricht the Netherlands; ^6^ MHeNs, School for Mental Health and Neuroscience, Maastricht University Maastricht the Netherlands; ^7^ Faculty of Psychology and Neuroscience, Department of Cognitive Neuroscience Maastricht University Maastricht the Netherlands; ^8^ Faculty of Psychology and Neuroscience, Department of Neuropsychology and Psychopharmacology Maastricht University Maastricht the Netherlands

**Keywords:** cognition, cognitive impairment, executive function, insulin resistance, maternal health, metabolic syndrome, obstetrical complication, postpartum, preeclampsia

## Abstract

**Objective:**

Preeclampsia contributes to maternal cognitive problems, particularly involving executive functions. These higher‐order cognitive functions—including working memory, organisation of materials, and task focus—are essential for adaptive, purposeful, and goal‐directed behaviour. Similar cognitive problems are observed in metabolic syndrome and insulin resistance. This study investigates whether these conditions are also associated with executive function after preeclampsia.

**Design:**

Nested case–control study.

**Setting:**

Maastricht University Medical Centre+, a tertiary care hospital.

**Population:**

Women 0.5 to 30 years after preeclampsia.

**Methods:**

The Behaviour Rating Inventory of Executive Function for Adults provided a measure of executive function performance. The National Cholesterol Education Program Adult Treatment Panel III defined metabolic syndrome. The Homeostatic Model Assessment for Insulin Resistance (HOMA‐IR) quantified insulin resistance. Participants were matched on age, postpartum time, and educational attainment. Associations of attenuated executive function with metabolic syndrome, its constituents, and insulin resistance were examined with conditional logistic regression.

**Main Outcome Measures:**

Odds ratios and population attributable fractions for the associations of attenuated executive function with metabolic syndrome, its constituents, and insulin resistance.

**Results:**

In 155 matched pairs, attenuated executive function was associated with metabolic syndrome (odds ratio 4.20 (95% confidence interval 1.58–11.14)), hyperglycaemia (2.96 (1.13–7.79)), and obesity (3.86 (2.00–7.47)). Attenuated executive function related to HOMA‐IR (7.26 (3.75–14.07)), and was 13% (6%–20%) attributable to metabolic syndrome and 56% (49%–67%) to insulin resistance.

**Conclusions:**

Metabolic syndrome and insulin resistance are associated with attenuated executive function after preeclampsia. Our findings provide leads for future studies focused on improving post‐preeclamptic cognitive performance.

## 1 Introduction

Gestational hypertension affects one in ten pregnancies, with half of hypertensive pregnancies progressing into preeclampsia when signs of maternal organ damage, conventionally being proteinuria or restricted fetal growth, emerge [1]. Delivery of the placenta is essential to prevent further immediate complications, yet women remain at heightened risk for long‐term cardiovascular disease, including hypertension, heart failure, myocardial infarction, and stroke, necessitating timely cardiovascular risk management [2].

Apart from an increased cardiovascular disease risk, cognitive problems also occur more frequently after preeclampsia [3, 4]. Former preeclamptic women are more prone to develop dementia, particularly vascular dementia, before the age of 40 years [5, 6]. Compared with post‐normotensive pregnancy, cognitive difficulties are nine times more likely after preeclampsia and notably involve higher‐order cognitive functions that are necessary for adequate control of purposeful, goal‐oriented, and adapted behaviour [3, 7, 8]. These higher‐order cognitive functions are collectively referred to as executive function and are essential for planning, organising, decision‐making, memorising, and paying attention [7].

The pathophysiological mechanisms of cognitive impairment after preeclampsia are unknown. Time is an important factor with risks of attenuated executive function declining with age and time elapsed since the preeclamptic pregnancy [3]. Independent of gestational history, a higher educational attainment protects against cognitive disturbances, whereas obesity poses a considerable risk [3]. Obesity is, among hyperglycaemia, hypertension and dyslipidaemia, one of the diagnostic criteria of metabolic syndrome, in which insulin resistance is a major underlying factor [9]. Metabolic syndrome and its constituents all relate to cognitive issues outside the context of preeclampsia, but sustained hypertension following a gestational hypertensive disorder is notably implicated [10, 11]. Given that metabolic syndrome is a risk factor for preeclampsia, we hypothesise that metabolic syndrome contributes substantially to post‐preeclamptic cognitive performance [12]. Because metabolic syndrome constituents are modifiable through medication and lifestyle changes, such interventions may not only reduce cardiovascular risk but also mitigate cognitive dysfunction following preeclampsia.

In this study, we examined the associations of attenuated executive function with metabolic syndrome, its constituents, and insulin resistance in a population with a history of preeclampsia.

## 2 Methods

This nested case–control study is part of a cross‐sectional study which investigates subclinical cardiovascular disease after pregnancy (Heart Failure and Related Risk‐factors After Preeclampsia, Queen of Hearts [QoH] study; ClinicalTrials.gov Identifier: NCT02347540). The Dutch Heart Foundation funded the study (Hartstichting) (grant number 013T084). Patients were not involved in the study design, and a core outcome set was not available.

Eligible participants were aged ≥ 18 years and were between 0.5 and 30 years after the first preeclamptic pregnancy. Preeclampsia was defined as hypertension along with fetal growth restriction, proteinuria (≥ 300 mg per 24 h), or other signs of maternal organ damage after 20 weeks of gestation. Data were obtained during a single study visit between December 2014 and October 2019. We retrieved age, educational attainment, smoking and drinking habits, and obstetric and general medical history from medical records and patient interviews. Smokers were defined as participants who smoked at inclusion, and alcohol consumers as those who consumed at least 7 units weekly. Educational attainment was categorised as high, medium, and low according to the International Standard Classification of Education. Preterm delivery was defined as delivery before 37 weeks of gestation, and early‐onset preeclampsia was defined as disease onset before 34 weeks of gestation. Haemolysis, elevated liver enzymes, and low platelets (HELLP) syndrome was considered when lactate dehydrogenase ≥ 600 international units (IU)/L (Haemolysis), aspartate transaminase ≥ 70 IU/L (Elevated Liver enzymes), and platelets < 100 × 10^9^/L (Low Platelets). A birthweight below the 10th percentile of national birthweight charts defined small for gestational age [13].

Waist and hip circumferences, height, and weight (877, Seca, Hamburg, Germany) were measured. Body Mass Index (BMI) was calculated as weight in kilograms divided by height in meters squared. Median blood pressure and heart rate were recorded at rest for 30 min at 3‐min intervals in a sitting position using CARESCAPE V100 equipment from GE HealthCare Technologies Inc. (Chicago, Illinois, USA). Venous blood sampling was performed after an overnight fast to determine levels of high‐density lipoprotein (HDL) cholesterol (Cobas 8000 Roche, Basel, Switzerland), triglycerides (Cobas 8000 Roche, Basel, Switzerland), insulin (Immulite Xpi, Siemens Healthineers, Erlangen, Germany), and glucose levels (Cobas 8000 Roche, Basel, Switzerland).

Metabolic syndrome was diagnosed following the National Cholesterol Education Program Adult Treatment Panel III (NCEP ATP‐III) definition, which requires three or more of the following five criteria to be met: [9]

1. Hyperglycaemia: glucose ≥ 5.6 mmol/L or drug treatment for elevated blood glucose
2. Low HDL cholesterol: HDL < 1.3 mmol/L or drug treatment for low HDL cholesterol
3. Elevated triglycerides: triglycerides ≥ 1.70 mmol/L or drug treatment for elevated triglycerides
4. Obesity: waist circumference ≥ 88 cm
5. Hypertension: blood pressure ≥ 130/85 mmHg or drug treatment for hypertension

Insulin resistance was quantified using the Homeostatic Model Assessment for Insulin Resistance (HOMA‐IR), calculated as $\left(\text{fasting insulin}\;\left(\text{pmol}/L\right)\times \text{fasting glucose}\;\left(\text{mmol}/L\right)\right)/135$ and HOMA‐IR values ≥ 2.0 (≥ 75th percentile) defined insulin resistance [14, 15, 16].

Perceived executive function performance was assessed with the Dutch translation of the Behaviour Rating Inventory of Executive Function for Adults (BRIEF‐A) [7, 17]. Participants rated the frequency of 75 examples of executive function problems, such as difficulties with memory retention, performing multiple tasks simultaneously, and sustained concentration, on a 3‐point scale from 1, *never*, to 3, *often*. Missing values were substituted by 1, *never*, if permissible per BRIEF‐A manual; otherwise, the participant was excluded [7]. Frequency scores were converted to standardised T‐scores using the Dutch BRIEF‐A normative reference chart. As defined in the BRIEF‐A manual, the sum of scores reflects global executive function performance, and T‐scores ≥ 65 indicate attenuated executive function of clinical relevance [7].

The studied population was divided into groups with and without attenuated executive function. Participants were matched to control for confounding. Matching criteria included age within a 5‐year range, postpartum time within a 1‐year range, and high educational attainment. Statistical tests appropriate for matched case–control studies included McNemar's test for categorical data, Wilcoxon signed rank test for non‐normally distributed continuous data, and paired T‐test for normally distributed continuous data. *p*‐values were adjusted for multiple comparisons using the Benjamini‐Hochberg procedure with a false discovery rate set at 0.05. Statistical significance was defined at *p*‐values < 0.05.

We evaluated associations of attenuated executive function after preeclampsia with metabolic syndrome (model A), metabolic syndrome constituents (model B), obesity, hyperglycaemia, and HOMA‐IR (model C), and HOMA‐IR (model D). Odds ratios (ORs) and population attributable fractions (PAFs), which reflect the proportion of attenuated executive function in the population attributable to each metabolic constituent, were calculated with 95% confidence intervals (95% CIs) using conditional logistic regression. The assumptions of logistic regression were checked: the study design ensured independence of observations, variance inflation factors < 5 were considered acceptable, studentized residuals ≥ 3 identified potential outliers, and linearity in the logit was assessed with the Box‐Tidwell procedure. To evaluate model performance, we calculated the Bayesian Information Criterion (BIC) which balances data fit and model complexity. Lower BIC scores indicate superior model performance. The association of attenuated executive function with HOMA‐IR was further analysed through recursive partitioning. Analyses were run in IBM SPSS Statistics (version 29, 2022) and R Statistical Software (version 4.3.3).

## 3 Results

Out of 1085 participants with a history of preeclampsia, 1036 (95%) provided valid BRIEF‐A responses and, among those, 1008 (97%) participants completed all measurements necessary for metabolic syndrome diagnosis. 155 participants reported attenuated executive function of clinical relevance. Sample characteristics are presented in Table 1. No statistically significant differences in age, smoking and drinking habits, educational attainment, and obstetric characteristics were observed between our groups with attenuated and normal executive function.

**TABLE 1:** Sample characteristics.

Characteristic	Attenuated executive function	Normal executive function	*p*
Number of participants	155	155	
Age, years	35.5 (6.64)	35.6 (6.12)	0.80
Intoxications			
Smoker	13 (8%)	12 (8%)	1.00
Alcohol consumer	5 (3%)	9 (6%)	0.39
Educational attainment			0.58
High	68 (44%)	68 (44%)	
Average	76 (49%)	79 (51%)	
Low	11 (7%)	8 (5%)	
Obstetric history			
Primiparous	86 (55%)	83 (54%)	0.76
Time postpartum, years	3.0 (1.17–7.00)	3.1 (0.92–7.17)	0.92
Caesarean delivery	66 (43%)	70 (45%)	0.74
Multiple gestation	8 (5%)	9 (6%)	1.00
Birthweight percentile	7 (2–29)	11 (4–33)	0.45
Small for gestational age	87 (56%)	72 (46%)	0.16
Gestational age, weeks^+days^	35^+0^ (32^+0^–37^+0^)	35^+4^ (33^+1^–37^+3^)	0.09
Preterm delivery, < 37 weeks	112 (72%)	99 (64%)	0.14
Perinatal mortality	9 (6%)	5 (3%)	0.42
Early‐onset preeclampsia	84 (54%)	76 (49%)	0.32
HELLP syndrome	109 (70%)	94 (61%)	0.08
Eclampsia	9 (6%)	15 (10%)	0.31
Recurrent preeclampsia	19 (12%)	15 (10%)	0.56

*Note:* Data are presented as number (percentage) of participants exhibiting the characteristic, mean (standard deviation), or median (interquartile range).

Abbreviation: HELLP, haemolysis, elevated liver enzymes and low platelets.

Metabolic characteristics are presented in Table 2. Metabolic syndrome was more prevalent in our sample with attenuated executive function (*n* = 26, 17%) compared with our sample with normal executive function (*n* = 10, 6%). Participants with attenuated executive function had lower HDL cholesterol and higher triglycerides, glucose, insulin levels, HOMA‐IR, weight, BMI, waist circumference, hip circumference, and waist‐to‐hip ratio. Blood pressure was comparable between both groups. Drug treatment for elevated glucose, low HDL cholesterol, elevated triglycerides, and hypertension was absent. Among participants with attenuated executive function, hyperglycaemia (*n* = 29, 19%), low HDL cholesterol (*n* = 41, 26%) elevated triglycerides (*n* = 16, 10%) and obesity (*n* = 83, 54%) were more prevalent compared with those with normal executive function (*n* = 10, 6%; *n* = 25, 16%; *n* = 5, 3%; and *n* = 36, 23%, respectively). Hypertension was comparably prevalent in our groups with attenuated executive function (*n* = 36, 23%) and normal executive function (*n* = 27, 17%). Participants with attenuated executive function (*n* = 85, 55%) were more frequently insulin resistant compared with those with normal executive function (*n* = 3, 2%).

**TABLE 2:** Metabolic syndrome characteristics.

Measure	Attenuated executive function	Normal executive function	Adjusted *p*
Metabolic syndrome	26 (17%)	10 (6%)	0.005
Hyperglycaemia	29 (19%)	10 (6%)	0.003
Glucose, mmol/L	5.2 (0.5)	5.0 (0.9)	0.044
Diabetes mellitus	1 (1%)	2 (1%)	1.00
Glucose lowering medication	0 (0%)	0 (0%)	—
Low HDL cholesterol	41 (26%)	25 (16%)	0.038
HDL cholesterol, mmol/L	1.5 (0.4)	1.7 (0.4)	< 0.001
HDL lowering medication	0 (0%)	0 (0%)	—
Elevated triglycerides	16 (10%)	5 (3%)	0.033
Triglycerides, mmol/L	0.88 (0.66–1.15)	0.72 (0.59–0.96)	0.003
Triglycerides lowering medication	0 (0%)	0 (0%)	—
Obesity	83 (54%)	36 (23%)	< 0.001
Weight, kg	75 (16)	65 (10)	< 0.001
Height, cm	168 (6)	168 (7)	0.98
BMI, kg/m^2^	27 (6)	23 (3)	< 0.001
Waist circumference, cm	90 (13)	81 (9)	< 0.001
Hip circumference, cm	106 (11)	99 (9)	< 0.001
Waist‐to‐hip ratio	0.81 (0.78–0.86)	0.79 (0.74–0.84)	< 0.001
Hypertension	36 (23%)	27 (17%)	0.25
Systolic blood pressure, mmHg	116 (13)	114 (11)	0.11
Diastolic blood pressure, mmHg	72 (9)	72 (9)	0.84
Antihypertensive medication	0 (0%)	0 (0%)	
Insulin resistance	85 (55%)	3 (2%)	< 0.001
Insulin, pmol/L	54.7 (33.9–83.2)	22.8 (15.2–30.0)	< 0.001
HOMA‐IR	2.1 (1.2–3.1)	0.82 (0.55–1.1)	< 0.001

*Note:* Data are presented as numbers (percentage) of participants exhibiting the characteristic, means (standard deviation), or medians (interquartile range). *p*‐values are adjusted for multiple comparisons using the Benjamini‐Hochberg procedure.

Abbreviations: BMI, body mass index; HDL, high‐density lipoprotein; HOMA‐IR, homeostatic model assessment of insulin resistance.

**FIGURE 1:**
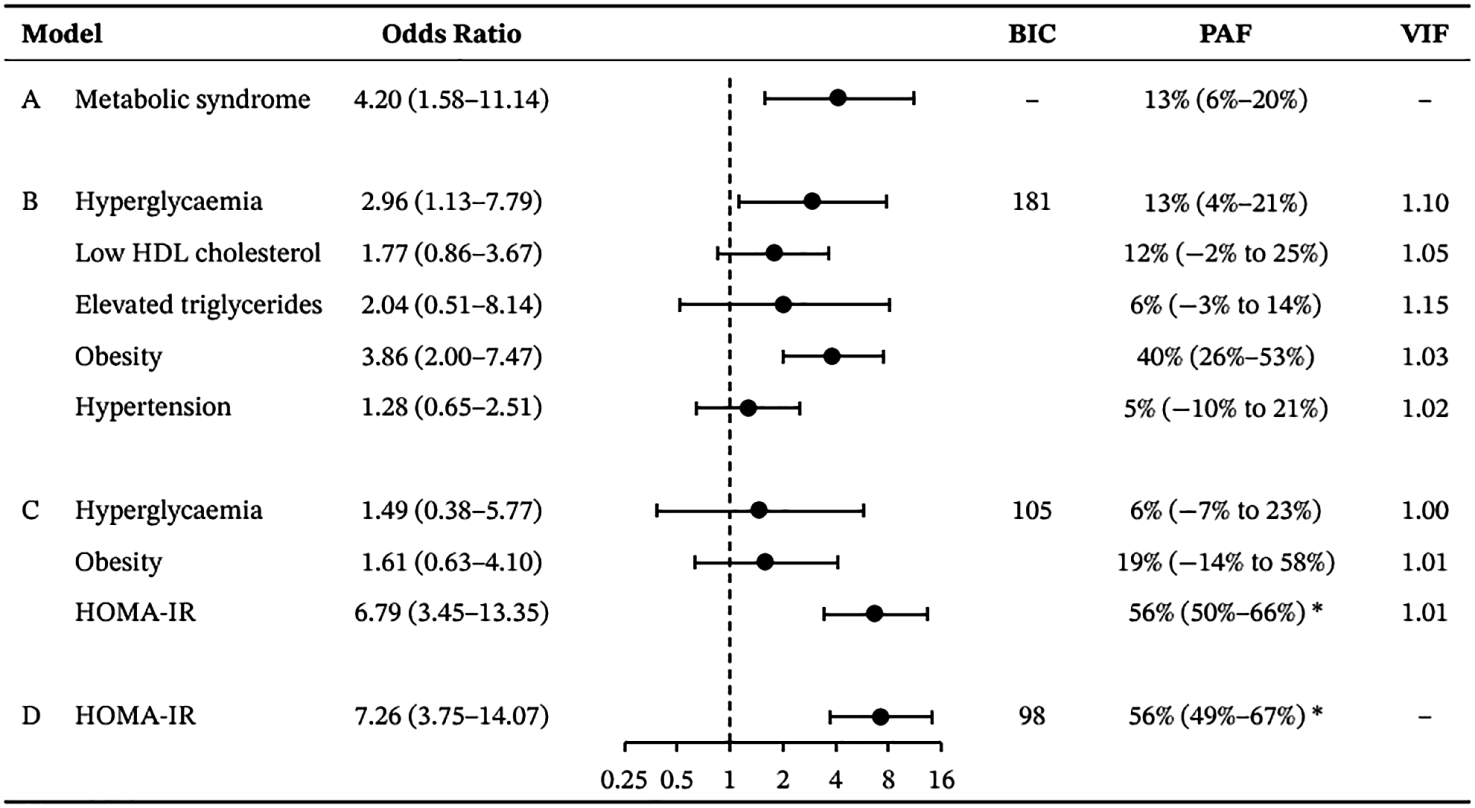
Odds ratios and population attributable fractions of reporting Behaviour Rating Inventory of Executive Function for Adults (BRIEF‐A) determined clinically relevant attenuated executive function in relation to the presence of metabolic syndrome (Model A), presence of metabolic syndrome constituents (model B), presence of hyperglycaemia, obesity and Homeostatic Model Assessment of Insulin Resistance (HOMA‐IR) (model C), and HOMA‐IR (model D). Odds ratios are presented as odds ratio (95% confidence interval) and population attributable fractions (PAF) as PAF (95% confidence interval). Abbreviations: BIC, Bayesian information criterion; HDL, high‐density lipoprotein; HOMA‐IR, homeostatic model assessment of insulin resistance; PAF, population attributable fraction; VIF, variance inflation factor. *PAF was calculated for the 75th HOMA‐IR (≥ 2.0) percentile defining insulin resistance.

**FIGURE 2:**
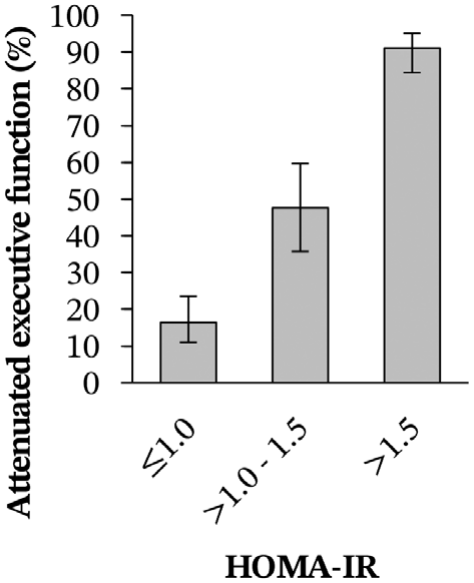
Fractions of Behaviour Rating Inventory of Executive Function for Adults (BRIEF‐A) determined clinically relevant attenuated executive function (%) along Homeostatic Model Assessment for Insulin Resistance (HOMA‐IR) values after recursive partitioning. Error bars represent 95% confidence intervals.

Multicollinearity, outliers, and nonlinearity in the logit were not detected in our regression models. As shown in Figure 1, model A had the largest BIC value and model D the smallest. In model A, the OR between attenuated executive function and metabolic syndrome was 4.20 (95% CI, 1.58–11.14), and 13% (95% CI, 6%–20%) of our sample reporting attenuated executive function was attributable to metabolic syndrome. In model B, attenuated executive function associated strongly with hyperglycaemia (OR 2.96 [95% 1.13–7.79], PAF 13% [95% CI, 4%–21%]) and obesity (OR 3.86 [95% CI, 2.00–7.47], PAF 40% [95% CI, 26%–53%]). Hypertension, low HDL cholesterol, and elevated triglycerides had no substantial contribution. In model C, which also included HOMA‐IR, the effects of hyperglycaemia (OR 1.49 [95% CI, 0.38–5.77], PAF 6% ([95% CI, −7% to 23%]) and obesity (OR 1.61 [95% CI, 0.63–4.10], PAF 19% [95% CI, −14% to 58%]) were twice as small compared with model B. Attenuated executive function was most strongly associated with HOMA‐IR, with an OR of 6.79 (95% CI, 3.45–13.35) per unit increase in HOMA‐IR score. In model D, each unit increase in HOMA‐IR was associated with a 7.26‐fold (95% CI, 3.75–14.07) increase in the odds of attenuated executive function, corresponding to a PAF of 56% (95% CI, 49%–67%) for insulin resistance. As shown in Figure 2, attenuated executive function increased proportionally across increasing levels of HOMA‐IR ranging from 18% (95% CI, 12%–25%) when HOMA‐IR ≤ 1.0 to 91% (95% CI, 84%–95%) when HOMA‐IR > 1.5.

## 4 Discussion

### 4.1 Main Findings

**FIGURE 3:**
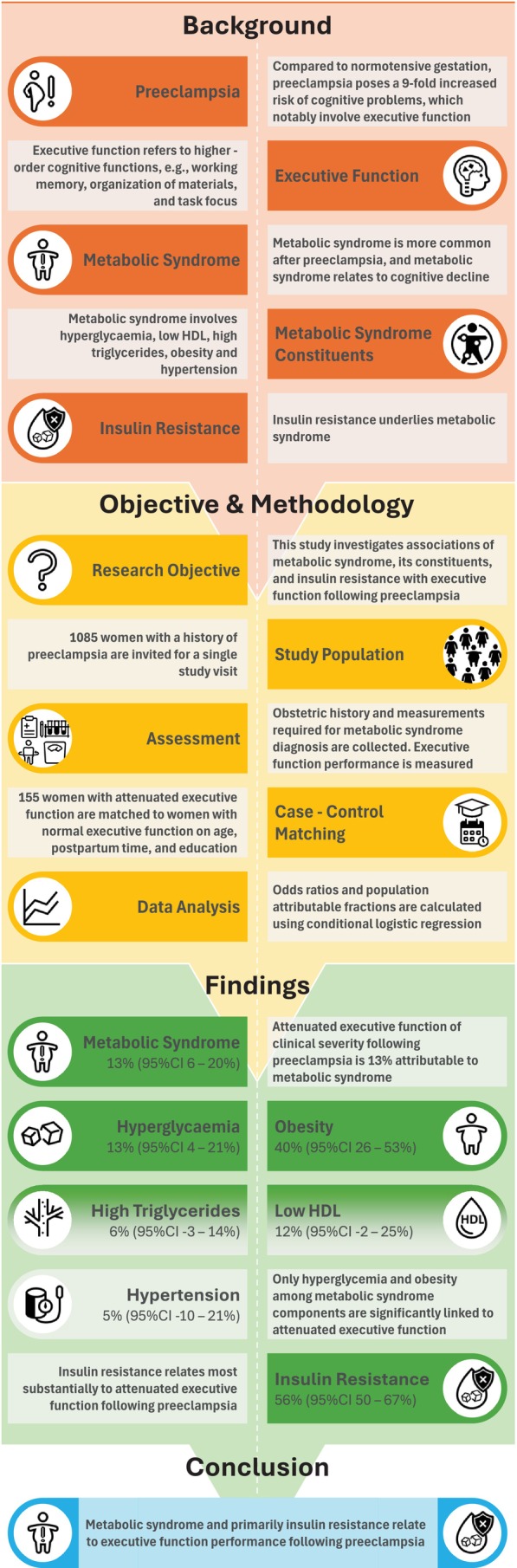
Graphical abstract of study rationale, methods, and key findings.

Metabolic syndrome is associated with attenuated executive function after preeclampsia in our studied population. Of the metabolic syndrome constituents, hyperglycaemia and obesity are particularly involved. Our findings, including the observed strong association with HOMA‐IR, support a prominent role for insulin resistance in attenuated executive function following preeclampsia. Figure 3 provides a graphical abstract summarising our methods and key findings.

### 4.2 Strengths and Limitations

This study has several strengths that merit consideration. First, we recorded executive function performance with a clinically valid self‐report that captures cognitive difficulties that truly impact daily life activities. Second, metabolic factors are examined in conjunction with executive function performance. As metabolic factors and executive function performance are subject to time, simultaneous assessment is required to reliably examine potential associations. Third, confounding is addressed by matching participants on age, time elapsed after preeclampsia, and educational attainment, resulting in a relatively young sample. The observed associations may contribute to the development of future therapeutic regimens aimed at improving executive function during the prime of life and preventing further cognitive decline. Fourth, metabolic syndrome definitions vary in scientific literature and occasionally involve surrogate markers. We applied the most used definition to diagnose metabolic syndrome (NCEP ATP‐III) without criterion modifications to facilitate comparisons with existing and future research.

Some limitations should be addressed. First, consensus regarding a cutoff for biological markers to identify insulin resistant individuals is lacking [15]. We defined insulin resistant as HOMA‐IR values at or above the 75th percentile. This cutoff corresponds to the World Health Organisation's definition of the top quartile as insulin resistant, based on insulin‐stimulated glucose uptake in clamp studies [15, 16]. To address the potential loss of statistical power associated with dichotomization, our data is analysed both categorically and continuously. The outcomes are comparable, which further substantiates the validity of our findings. Second, our study population was drawn from a larger study examining cardiovascular health after preeclampsia and normotensive pregnancy. Although participation may have been influenced by interest in long‐term health risks, potentially leading to a studied population with higher educational attainment, selection bias on cognitive complaints is minimised while the potential confounding effect of educational attainment is methodologically controlled for [18]. Third, participants were eligible for study participation up to 30 years after preeclampsia, which could affect the accuracy of retrieved obstetric history. To ensure optimal data quality, trained interviewers obtained data from medical records and supplemented data anamnestically. Fourth, because no participants received treatment for any metabolic condition, we could not assess the potential therapeutic effect of such treatments on cognitive performance. Finally, we can only report associations as data regarding metabolic syndrome and cognitive status prior to preeclampsia are lacking. Therefore, we recommend longitudinal and interventional follow‐up studies to investigate causality.

### 4.3 Interpretation

Our work reveals important associations between executive function and metabolic syndrome, its constituents (except for hypertension), and insulin resistance in a population with a history of preeclampsia. Consistent with this, metabolic syndrome, its constituents, and insulin resistance relate to reduced executive function performance in the general female population [10, 19]. Because cognitive decline is more pronounced the longer hypertension persists, the observed absence of an association with hypertension likely results from a lower cumulative blood pressure load due to the relatively young age of our studied population [20, 21].

The mechanisms underlying the relationships between executive function, metabolic syndrome, and insulin resistance after preeclampsia are unknown. We have described the impact of preeclampsia on executive function and its clinically derived scales in earlier work [3]. The observed difficulties relate particularly to emotional regulation and working memory and suggest involvement of the prefrontal cortex and hippocampus [3]. These brain regions notably rely on insulin signalling to support cognitive processes by maintaining neuronal survival, density, plasticity, and connectivity [22, 23]. While it is highly speculative to link peripheral glucose and metabolic derangements to cognitive performance after preeclampsia, our findings uncover important associations between executive function and obesity, hyperglycaemia, and insulin resistance.

Obesity and diabetes mellitus type 2 are known risk factors for cerebral atrophy and cognitive difficulties, with global cerebral atrophy and especially hippocampal atrophy being important predictors of executive function decline [24, 25]. Because global and regional cerebral volumes are similar after preeclampsia compared to after normotensive pregnancy, we consider brain atrophy unlikely to be responsible for cognitive difficulties after preeclampsia [26].

Blood–brain barrier integrity together with insulin resistance may be pivotal biological elements in the observed epidemiological relationship between executive function performance and a history of preeclampsia. Endothelial dysfunction is typical for preeclampsia, and blood–brain barrier leakage rates remain elevated many years after preeclampsia [27]. Metabolic syndrome constituents raise levels of reactive oxygen species that damage the vascular endothelium, including that of the blood–brain barrier, and cause endothelial dysfunction [28]. The resulting disruption of cerebrovascular integrity may inflict cognitive difficulties. The endothelial cells lining the blood–brain barrier facilitate adequate insulin transport across the blood–brain barrier, and loss of blood–brain barrier integrity may hamper insulin transport and cause subsequent cognitive difficulties [29]. Disruption of cerebral insulin signalling harms cognitive functions in mice [23]. In our studied population, reduced insulin sensitivity relates to a higher prevalence of attenuated executive function even among participants with HOMA‐IR values below the 75th percentile threshold, indicating a low level of insulin resistance. Considering the compromised blood–brain barrier integrity that persists years following preeclampsia and the observed relationship between executive function and insulin resistance, we hypothesise that dysregulated cerebral insulin signalling contributes to the executive function difficulties observed after preeclampsia [27].

From a therapeutic perspective, the involvement of insulin resistance with attenuated executive function in our sampled population suggests that existing treatments may be helpful. Maintaining a healthy body composition is recommended to improve health in general and to reduce the risk of developing insulin resistance and cardiovascular disease. Weight loss improves cognitive performance, insulin sensitivity, and insulin transport across the blood–brain barrier [30, 31, 32]. As attenuated executive function relates substantially to obesity, weight loss may be particularly helpful for individuals with cognitive dysfunction after preeclampsia. Whether blood–brain barrier damage after preeclampsia resolves with concomitant cognitive improvement through weight loss remains to be determined.

Weight loss is achievable through physical exercise and diet. Physical exercise strengthens cognitive processes by organising and creating new synaptic connections [33, 34]. Together with dietician‐guided dieting, exercise improves cerebral glucose metabolism and insulin sensitivity [35]. A low‐fat diet also lowers the risk of cognitive difficulties [36]. Given that attenuated executive function relates univariably to high triglycerides and low HDL cholesterol in our studied population (Table 2), physical exercise combined with a low‐fat diet may improve executive function performance by enhancing functional connectivity within the prefrontal cortex and limbic system [37]. A low‐fat diet likely exerts its beneficial effect via weight loss and concomitant improved insulin sensitivity, instead of lowering cholesterol levels, based on our multivariable analysis of metabolic syndrome constituents (Figure 1, model B).

Weight loss is the most desirable approach from a health and cost perspective, but it is a slow and difficult strategy with only one in five adults being able to maintain weight loss long‐term [38]. Pharmacological interventions to improve cerebral insulin sensitivity may be explored. For example, intranasally administered insulin improves cognitive functions [39]. Assuming brain insulin resistance plays a pivotal role in executive function, increasing cerebral insulin sensitivity and availability may improve cognitive performance after preeclampsia. Alternatively, hypoglycaemic agents show mixed results regarding cognition, yet therapies using glucagon‐like peptide‐1 receptor agonists appear consistently beneficial [40]. Considering the observed associations of obesity and hyperglycaemia with executive function post‐preeclampsia, glucagon‐like peptide‐1 receptor agonists semaglutide and liraglutide are particularly promising because of their approval in the treatment of obesity and diabetes mellitus type II [41]. Whether intranasally administered insulin and hypoglycaemic agents improve executive function after preeclampsia requires further research.

## 5 Conclusion

In conclusion, attenuated executive function of clinical relevance after preeclampsia is four times more likely in the presence of metabolic syndrome. Insulin resistance accounted for 56% of the population attributable fraction for attenuated executive function. These findings help clinicians recognise women at risk of cognitive dysfunction and guide targeted research into future treatment regimens.

## Author Contributions

C.G.‐D. and M.E.A.S. conceptualised and designed the study. R.‐J.A. wrote the first draft of the paper. All authors revised and contributed to the intellectual content of the manuscript.

## Ethics Statement

The Medical Ethics Committee of the Maastricht University Medical Centre+ (MUMC+) in the Netherlands (METC azM/UM 14‐2‐013 NL47252.068.14) granted ethical approvals and methods adhered to the principles of the Declaration of Helsinki.

## Conflicts of Interest

The authors declare no conflicts of interest.

## Data Availability

The data that support the findings of this study are available from M.E.A.S. upon reasonable request.
